# Effects of disturbance on vegetation by sand accretion and erosion across coastal dune habitats on a barrier island

**DOI:** 10.1093/aobpla/plv003

**Published:** 2015-01-12

**Authors:** Thomas E. Miller

**Affiliations:** Department of Biological Science, Florida State University, Tallahassee, FL 32306, USA

**Keywords:** Climate change, coastal zones, disturbance, geomorphology, ordination, plant community

## Abstract

Coastal geomorphology and vegetation are expected to be particularly sensitive to climate change, because of disturbances caused by sea-level rise and increased storm frequency. Dunes have critical reciprocal interactions with vegetation; dunes create habitats for plants, while plants help to build dunes and promote geomorphological stability. These interactions are also greatly affected by disturbances associated with sand movement, either in accretion (dune building) or in erosion. The magnitude and intensity of disturbances are expected to vary with habitat, from the more exposed and less stable foredunes, to low-lying and flood-prone interdunes, to the protected and older backdunes. Permanent plots were established at three different spatial scales on St George Island, FL, USA, where the vegetation and dune elevation were quantified annually from 2011 to 2013. Change in elevation, either through accretion or erosion, was used as a measure of year-to-year disturbance over the 2 years of the study. At the scale of different dune habitats, foredunes were found to have the greatest disturbance, while interdunes had the least. Elevation and habitat (i.e. foredune, interdune, backdune) were significantly correlated with plant community composition. Generalized linear models conducted within each habitat show that the change in elevation (disturbance) is also significantly correlated with the plant community, but only within foredunes and interdunes. The importance of disturbance in exposed foredunes was expected and was found to be related to an increasing abundance of a dominant species (*Uniola paniculata*) in eroding areas. The significant effect of disturbance in the relatively stable interdunes was surprising, and may be due to the importance of flooding associated with small changes in elevation in these low-lying areas. Overall, this study documents changes in the plant community associated with elevation, and demonstrates that the foredune and interdune communities are also associated with the responses of specific species to local changes in elevation due to accretion or erosion.

## Abstract (precis)

Understanding how plants stabilize coastal dunes will be important for predicting effects of climate change along shorelines. This study used three years of data on plant communities and dune morphology on a barrier island to show how particular plant communities were associated with the disturbances that occur in coastal dunes. As expected, foredunes were very disturbed, with important effects on their vegetation. However, the much smaller disturbances that occurred in areas behind the foredunes had similar effects on the plant communities. These results will be important for modeling how increased storms and ocean level rise will affect sandy coasts.

## Introduction

Coastal sand dunes provide the first line of defense against storms and high water levels in many parts of the world (Sallenger 2000; Ruggiero *et al*. 2001; Feagin *et al.* 2005). As such, coastal ecosystems are particularly sensitive to sea-level rise and any changing frequencies of tropical storms and hurricanes, all of which are predicted to occur with global climate change (e.g. Duran and Moore 2013; Prisco *et al.* 2013). These climate effects may be especially important because dunes have a high ecosystem value as habitat for endemic plants and animals, while sheltering bay (e.g. seagrass, oyster beds and saltmarsh) habitats, as well as inland wetlands and marshes (Martinez and Psuty 2004; Gutierrez *et al.* 2011). Dunes can also be important for protecting coastal towns, as well as the economic activities they provide (e.g. fisheries, tourism).

The development and maintenance of coastal dune habitats requires a plentiful supply of sand, strong winds to move sand inland and an obstacle, usually plants, to stop the sand and create dunes. Thus the plants on dunes have long been recognized as key components of coastal habitats. The vegetation on dunes has served as a model system for influential studies of plant succession and ecology (Cowles 1899; Oosting and Billings 1942); because dunes form near shore, dunes that are progressively inland create chronosequences for vegetation studies.

The relationship between plants and dunes is both reciprocal and complex (e.g. Stallins and Parker 2003). The plants are thought to control sand movement and determine the shape and position of the dunes (e.g. Moreno-Casasola 1986), while the dune structure can determine the abiotic factors such as soil moisture and nutrients that control plant establishment, growth and reproduction (Ehrenfeld 1990). Many previous studies of coastal dune vegetation identified harsh physical factors such as salt spray, soil moisture and sand movement as the primary factors responsible for the zonation patterns parallel to the beach (e.g. Oosting and Billings 1942; Miller *et al.* 2010; Bitton and Hesp 2013).

Fewer studies have considered the feedbacks between plants and the dune geomorphology. It has been shown that certain species, frequently grasses, are correlated with dune formation on beach plateaus, generally promoting the development of foredunes (Gibson and Looney 1994; Stallins and Parker 2003; Bitton and Hesp 2013). Dune building plants have also been shown to slow moving sand particles (Zarnetske *et al.* 2012) and to be highly tolerant of burial and, for marine coasts, high salinity. But, less is known about how plants influence the continued growth and maintenance of foredunes, or how they affect more inland areas such as interdunes (also called overwash plateaus) and older backdune areas. For example, some plants have been associated with dune accretion (Bitton and Hesp 2013), while other plants may hinder dune formation, promoting lower and flatter areas of interdunes (Ehrenfeld 1990; Wolner *et al.* 2013). But, almost nothing is known of interactions between vegetation and geomorphology across different spatial scales (e.g. habitats) in coastal dunes.

This lack of knowledge about dune processes at various spatial scales makes it difficult to predict the effects of climate change on sandy coastal habitats. The short- and long-term effects of both sea-level rise and increased storm frequencies are relatively unknown, but are expected to be significant, given the low elevation and dynamic nature of the geomorphology of coastal dunes. Understanding the effects of the disturbance caused by sand accretion or erosion on dune plant communities will help to elucidate the mechanisms by which global climate change can affect areas such as barrier islands, sandy bars and spits. Ultimately, this should also help for predicting and preparing for the effects of climate change.

This study uses multi-year plant and elevation surveys of St George Island, FL, USA to (i) quantify the dynamic relationship between dune elevation and plant communities. The study also determines (ii) whether the nature of the relationships among elevation, change in elevation and the plant community changes with spatial location, as one moves from the newer and more disturbed foredunes to the low and wet interdunes, and finally to older, more protected backdunes.

## Methods

### Site description

St George Island, FL, USA (29°46′00″N, 84°41′30″) is typical of barrier islands that form with low tidal ranges on wave-dominated coasts. It is ∼45 km long and 1 km wide, off the Florida panhandle in the northern Gulf of Mexico. Such islands worldwide share a number of habitats maintained by the wind and wave forces that created the islands themselves. At the ocean side of the island, foredunes are created and maintained by the sand blown from the beach plateau. They have high (3–5 m) and dynamic dune ridges subject to wind, spray and high tides. Foredunes are lower in overall plant diversity and are frequently quite dry (Miller *et al.* 2010). Behind the foredunes are interdunes, which are low, and relatively flat and homogeneous in elevation. The sand in the interdune contains more organic material than other dune areas and is often wet or flooded. Saltwater will inundate interdunes with major storms, but they more frequently fill with freshwater from rains as the lens under the island fills. Finally, further inland from the interdunes are backdunes, which consist of irregular but stable ridges (1–2 m height) separated by troughs and can extend to the bay side of the island. Backdunes are much more stable than foredunes and have the highest plant diversity (Miller *et al.* 2010). They contain some woody species, as well as species also common to both interdunes and foredunes. Older and wider islands may also include later stages of dune and vegetation succession, but these are not present at the relatively young field site used in this study.

### Sampling design

**Figure 1.**
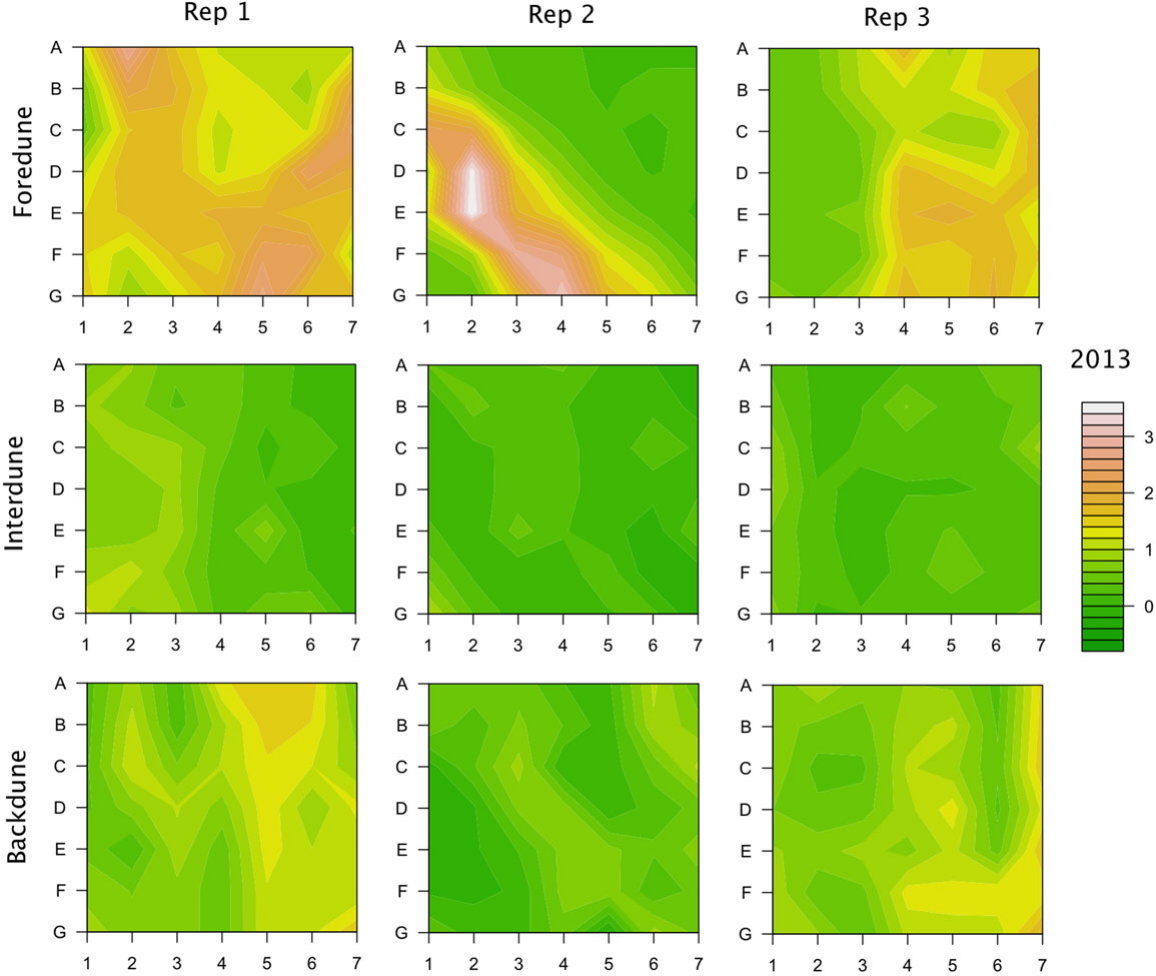
Topographic maps from 2013 of each of the nine grids used in this study. Grids are 60 × 60 m, with a 7 × 7 grid of points and each point 10 m from the next. The scale at right is in metres, and the 0 value is arbitrary. Extrapolated points were estimated using ‘filled.contour’ in R (R Core Team 2014).

This work was conducted in the St George Island State Park, which occupies the easternmost 14 km of the island. A study was established in 1999 to follow the vegetation on the actively growing eastern tip of St George Island and has been continued annually since (except for 2002; see Miller *et al.* 2010). Initially, two grids were set up in each of the three habitats (foredune, interdune and backdune); one more replicate grid was set up in each habitat in 2010. Each of the nine grids consists of 49 plots in a 7 × 7 array, with 10 m between plots. A wooden stake marks each plot and the vegetation in a 1 m^2^ area to the northeast of each stake is censused in the late fall of every year. The per cent cover of each species per plot is recorded, along with any special environmental factors such as sand disturbance or flooding. Elevations for each of the 441 plots were determined each fall from 2011 to 2013 using a rotating laser level (Topcon RL-H3C). No elevation ‘standard’ is available in this area as past hurricanes have uprooted benchmarks. Because of the unstable nature of the landscape, an average elevation of 98 stakes in backdune areas thought to be stable were used as a standard elevation to compare across years. The topographic differences among the three habitats can be clearly seen by looking at these elevations (Fig. 1).

### Data analysis

Data used in this paper are from the 3 years of 2011–13. To quantify the plant community in any given year, the per cent cover data were converted to the presence/absence of each species on each plot to minimize noise from the error associated with estimating per cent cover (i.e. Hirst and Jackson 2007). Then nonmetric multidimensional scaling (NMDS) was applied using the ‘vegan’ package of R (R Core Team 2014), with a maximum of 20 random starts in search of a stable solution. In general, stable solutions were not found, but repeated runs of metaMDS gave very similar results.

To minimize the effects of rare species, only species that were found on more than 5 % of the plots were included in the ordination. The effects of habitat and grid within habitat were evaluated using PERMANOVA through the ‘adonis’ function in the vegan package.

**Figure 2.**
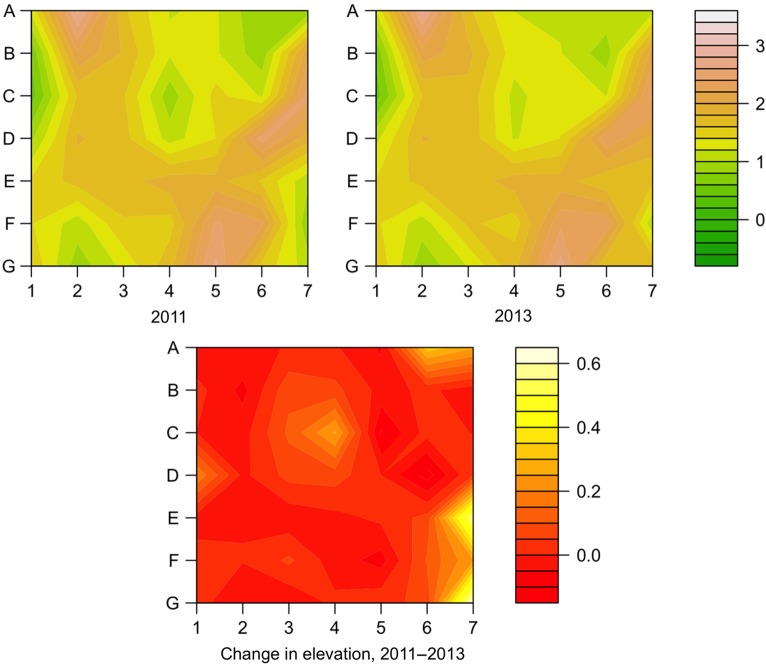
An example of the change in the topography of a foredune plot on St George Island from 2011 to 2013. The top two figures show the same plot in the first and last year of this study, with north towards the top. The lower figure shows the difference in elevation between the top two plots; note the much smaller scale. For this foredune plot, elevations are increasing on the eastern, more shoreward, side as winds carry sands up the beach plateau.

The elevations in 2011 and 2013 were compared to get the change in elevation over the 2-year span (see example in Fig. 2), showing either accretion or erosion (analyses by single-year spans found similar results). To show the effect of sand accretion or loss for the previous 2 years on plant community structure in 2013 while accounting for the effects of elevation, the vegetation in 2013 was characterized for each 1 m^2^ plot using the NMDS scores determined above, and then the first and second axes scores were used as the dependent variables in generalized linear models (glm function in R), with the independent variables of (i) habitat, (ii) elevation in 2011 and (iii) change in elevation between 2011 and 2013. Replicate grid ID did not have a significant contribution from the model for any habitat and was not included in the results presented here. The change in elevation has a very awkward distribution, with both extreme high and low outliers that could not be easily transformed. To allow the data to be analysed, only the extreme outliers were transformed, by assuming that all values >0.2 m were equal to 0.2 m and all less than −0.2 m were equal to −0.2 m. Because there were strong interactions between the effects of some of the independent variables, separate GLM analysis were then conducted within each habitat, using just elevation and change in elevation as variables. The loadings of individual species in the NMDS were then used to determine which individual species were contributing the most to overall patterns in each habitat.

To determine which species were most correlated with disturbance, individual species per cent cover were correlated with elevation and with change in elevation per plot within each habitat using non-parametric Kendall *τ* values. Because this involves many comparisons, *P* values were corrected for a false discover rate (Benjamini and Hochberg 1995).

## Results

**Figure 3.**
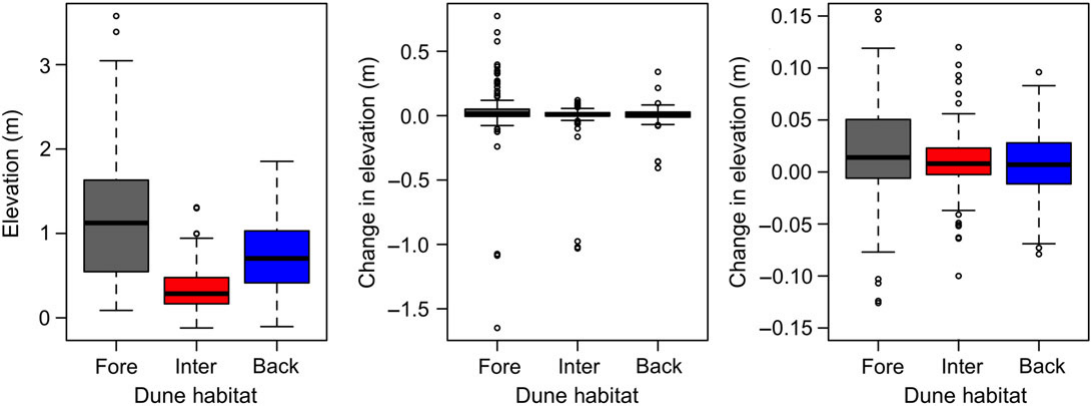
Boxplots showing the median (bar), 50 % confidence intervals (CI) (box) and 97 % CI (whiskers) for elevation (left) and change in elevation (centre and right) for the 147 plots in each habitat. The centre plot includes the outliers, while the right plot shows the same boxplots more closely, so the median and CI can be seen.

The elevation of the dunes varied over almost 4 m in these relatively young dunes (Fig. 3), with the highest areas along foredune ridges (Fig. 1). Foredunes were on average the highest and most variable of the three habitats, while interdunes were not surprisingly the lowest and least variable. The change in elevation from 2011 to 2013 was generally very small (Fig. 3), with notable exception of dunes gaining over 0.6 m or losing over 1.5 m. These were generally due to the collapse of foredune ridges into leeward areas more inland. This caused a loss of dune elevation along the ridge but an increase in elevation in the lower areas were the sand ended up.

**Figure 4.**
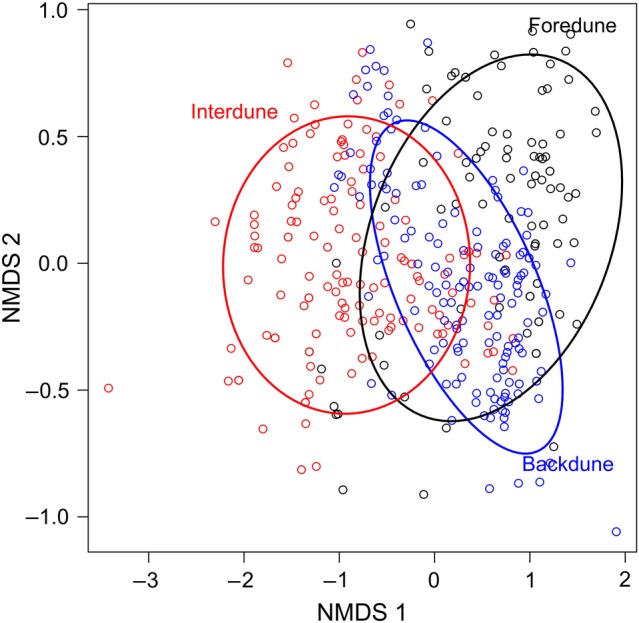
Ordination of the vegetation on 441 plots on St George Island, from 2013, using NMDS (stress = 0.112). Ovals denote ∼75 % CI for the 147 plots found in each of the three habitats, foredunes (black), interdunes (red) and backdunes (blue).

Over 60 species have been documented across the three dune habitats, with species richness increasing from foredunes to interdunes to backdunes (Miller *et al.* 2010). Vegetation on the dunes also varied among habitats, as shown by the NMDS (Fig. 4), with a significant effect of habitat (*F* = 56.1, *P* < 0.001) and replicate grid nested within habitat (*F* = 10.1, *P* < 0.001). As noted in Miller *et al.* (2010), interdunes are generally dominated by species associated with wetter areas, such as *Juncus* spp., *Phyla nodiflora* and *Paspalum distichum*, while foredunes and backdunes have species associated with drier areas, such as *Uniola paniculata*, *Schizachyrium maritima* and *Ipomoea imperata*.

The analysis of the full model predicting NMDS scores for 2013 based on habitat, elevation in 2011 and change in elevation from 2011 to 2013 suggested that there were significant effects of elevation, habitat and change in elevation, with elevation particularly loading on the first NMDS axis (Table 1). However, because of the significant interactions, especially between elevation and habitat, the data must be analysed separately for each habitat to determine the effects of elevation and change in elevation.

**Table 1.** *F*-values from generalized linear models of the effects of dune elevation and change in elevation from 2011 to 2013 on NMDS axis scores for vegetation on St George Island, from 2013 (**P < 0.01, ***P < 0.001). See text for details.

	NMDS axis 1	NMDS axis 2
Elevation	1177.70***	3.01
Habitat	136.05***	116.27***
Change in elevation	32.616***	9.89***
Elevation × habitat	55.149***	9.78***
Elevation × change in elevation	0.68	0.002
Habitat × change in elevation	16.09***	9.68***
Elevation × habitat × change in elevation	5.49**	0.08

The NMDS analysis of the separate dune habitats also confirmed that elevation was a major contributor to plant community structure across foredunes, interdunes and backdunes (Table 2). However, the change in elevation was significant only in foredune and interdune habitats; only elevation significantly contributed to NMDS scores in backdunes. There were generally no significant interactions between elevation and change in elevation, except in interdunes for the first NMDS axis.

**Table 2.** *F*-values from generalized linear models of the effects of elevation and change in elevation from 2011 to 2013 by habitat on NMDS axis scores for vegetation on St George Island in 2013 (*P < 0.05, **P < 0.01, ***P < 0.001). See text for details.

	Foredune		Interdune		Backdune	
	NMDS1	NMDS2	NMDS1	NMDS2	NMDS1	NMDS2
Elevation in 2011	133.68***	31.98***	390.36***	5.37*	271.938***	3.48
Change in elevation from 2011 to 2013	8.82**	18.17***	85.13***	6.34*	1.032	2.51
Elevation × change in elevation	0.133	1.77	15.7***	0.01	0.611	0.66

Finally, at the scale of the individual plots in each habitat, one can ask which species were significantly correlated with elevation or change in elevation and how. Several species were positively correlated with elevation across all habitats (Table 3), including most notably the grasses *Uniola paniculata* and *Schizachyrium maritimum*, as well as the forb *Ipomoea imperati*. Other species were negatively correlated with elevation across all three dune habitats, including *Phyla nodiflora*, *Eragrostis lugens* and *Fimbristylis spadacea*. However, this study particularly concerns species that were affected by changes in elevation. In the foredunes, the only species exhibiting a significant relationship with the change in elevation was the very abundant *Uniola paniculata*, which appears to increase in abundance as dune elevation decreases. In interdunes, the low-lying *Polypremum procumbens* was positively correlated with increasing elevation, while a species mostly found associated with standing water, *Polygonum punctatum*, was correlated with lowering elevations. In the backdunes, no species abundances were correlated with changes in elevation, which is consistent with the analyses of the ordination scores.

**Table 3.** Correlations between species per cent cover and elevation and species per cent cover and change in elevation, within each habitat. Analyses were done for species occurring in >5 % of the plots in that habitat. Values are Kendall *τ* values, with significance corrected for multiple comparisons (*P < 0.05, **P < 0.01, ***P < 0.001). NA, species not available.

Species	Foredune		Interdune		Backdune	
	Elevation	Change	Elevation	Change	Elevation	Change
*Bulbostylis ciliatifolia*	NA	NA	−0.03	0.06	−0.21**	−0.04
*Cenchrus incertus*	−0.05	−0.06	0.34***	−0.12	0.03	−0.03
*Centella asiatica*	−0.15	−0.00	−0.35***	0.10	−0.47***	−0.03
*Chamaesyce maculata*	−0.03	−0.01	NA	NA	0.04	−0.07
*Cnidoscolus stimulosus*	NA	NA	NA	NA	0.24**	0.03
*Cynanchum angustifolium*	−0.47***	0.04	−0.11	0.03	−0.36***	−0.12
*Cyperus croceus*	−0.09	0.11	0.09	0.03	0.10	0.05
*Rhynchospora colorata*	NA	NA	−0.16*	−0.02	−0.27***	0.04
*Eragrostis lugens*	−0.40***	0.03	−0.08	0.11	−0.41***	0.01
*Fimbristylis spadaceae*	−0.45***	−0.01	−0.11	0.08	−0.23**	0.04
*Fuirena scirpoidea*	NA	NA	−0.02	0.05	−0.15*	−0.05
*Heterotheca subaxillaris*	−0.07	−0.01	0.25**	0.02	0.11	−0.05
*Hydrocotyle bonariensis*	0.07	−0.06	0.12	−0.10	−0.16*	−0.13
*Ipomea imperati*	0.23**	0.01	0.12	−0.06	0.36***	0.01
*Iva imbricata*	0.22**	0.07	NA	NA	NA	NA
*Juncus megacephalus*	NA	NA	−0.07	−0.03	−0.15	0.07
*Muhlenbergia capillaris*	−0.16	0.06	0.09	0.12	−0.43***	−0.02
*Oenothera humifusa*	0.03	−0.15	0.41***	0.14	0.03	0.10
*Panicum aciculare*	NA	NA	0.08	0.00	−0.41***	−0.07
*Panicum amarum*	−0.07	0.03	0.20**	0.14	0.04	0.10
*Paronychia erecta*	NA	NA	NA	NA	−0.06	−0.16
*Paspalum vaginatum*	−0.30**	0.00	−0.48***	−0.12	NA	NA
*Physalis angustifolia*	NA	NA	0.33***	−0.05	0.04	−0.09
*Phyla nodiflora*	−0.47***	0.06	−0.40***	0.06	−0.22**	0.09
*Polypremum procumbens*	NA	NA	0.34***	0.20*	−0.19**	−0.07
*Polygonum punctatum*	NA	NA	0.10	−0.22*	NA	NA
*Schizachyrium maritimum*	0.31***	−0.13	0.50***	−0.07	0.30***	−0.05
*Sclerea verticillata*	NA	NA	−0.06	−0.03	−0.24**	0.02
*Setaria parvifolia*	NA	NA	NA	NA	−0.29***	−0.06
*Smilax auriculata*	NA	NA	0.09	0.11	0.15*	−0.02
*Spartina patens*	−0.05	0.12	−0.09	0.11	−0.17*	−0.08
*Sporobolus virginicus*	−0.12	−0.03	−0.01	0.13	−0.09	0.01
*Uniola paniculata*	0.45***	−0.20*	0.51***	−0.01	0.39***	0.03

## Discussion

Understanding how dunes and vegetation interact, especially following sand accretion and loss, will be critical for determining the vulnerability of coastal areas to climate change (e.g. sea-level rise and changing storm frequencies). The static patterns of dunes and vegetation have been well documented in a variety of coastal systems (e.g. Gibson and Looney 1994; Stallins and Parker 2003; Martinez and Psuty 2004). However, few studies have followed both dunes and vegetation through time in order to quantify the dynamic relationship between geomorphology and vegetation and no prior studies have been carried out at different scales across different dune habitats. This study demonstrates that the relationships among geomorphology, vegetation and disturbance vary in important ways in different parts of the dune ecosystem.

At the scale of the entire coastal ecosystem, elevation was the dominant factor correlated with vegetation patterns in 2013 (Table 1), consistent with other studies (e.g. Moreno-Casasola 1986; Bitton and Hesp 2013). However, foredunes, interdunes and backdunes can have very different types of vegetation (Fig. 4) and so habitat also explained a significant amount of variation in plant communities among plots. Despite these dominant effects of elevation and habitat, the amount of sand accretion or loss (disturbance) at each site also had a significant effect on the plant community structure, which has implications for the effects of climate change. Because it appears that the effect of elevation changes with dune habitat (Table 1), further analyses were conducted at the scale of the individual habitats.

At the scale of each dune habitat, the statistical models continue to show that elevation is the dominant factor correlated with vegetation patterns (Table 2). However, the change in elevation also has a significant correlation with vegetation patterns in the foredunes and interdunes, suggesting that vegetation in these habitats may be more affected by climate change than vegetation in the backdunes (Table 2). This is particularly interesting in light of the patterns of dune elevation and change in elevation across the three habitats (Figs 1 and 3). It is well known that foredunes tend to be taller and more subject to both dune building and erosion than other dune habitats, which is consistent with the patterns observed on St George from 2011 to 2013. So, it seems reasonable the higher sand movement, creating both increases and decreases in elevation, would affect the plant communities on foredunes. However, interdunes are the lowest and show the least variation in elevation over this time period. So, it is somewhat surprising that there is a correlation between vegetation pattern and change in elevation for interdunes. One possible explanation is that the lower-lying habitats are more affected by flooding; small changes in elevation can determine whether the plot is flooded or has saturated soil or is dry. Thus, small changes in elevation may have greater effects in interdunes than any other dune habitat, which also makes interdunes particularly susceptible to flooding associated with climate change.

At the scale of the individual species and plots, there are many significant correlations between the cover of particular species and elevation, as would be predicted from prior studies (e.g. Miller *et al.* 2010; Bitton and Hesp 2013; Wolner *et al.* 2013). These correlations are likely due to effects of soil moisture or soil nutrient levels, or both (see Miller *et al.* 2010). Some of the species showing the strongest associations with elevation are also among the most abundant species and characteristic of particular habitats and elevations. In particular, *U. paniculata* is considered important for stabilizing dune ridges; it is interesting that this species has the same association across all three dune habitats. *Schizachyrium maritima* has also been found to be an important species for restoration after hurricanes (Gornish and Miller 2013) and also favours higher elevation on dunes. *Iva imbricata* was also positively correlated with increasing dune elevation and is another species that may be important for stabilizing foredunes (see Colosi and McCormick 1978). Other species that increase in per cent with increasing elevation are *Oenothera humifusa* in interdunes (this species appears to be salt-intolerant; Miller *et al.* 2010), *Ipomoea imperata* and *Physalis angularis*, all of which can be relatively abundant on dune ridges in dune habitats.

Species that were negatively associated with elevation also include some relative dominants in each habitat. *Eragrostis lugens* and *Fimbristylis spadacea* are abundant in lower areas of foredunes and backdunes, but are generally not found in the wetter areas associated with interdunes. The grasses *Muhlenbergia capillaris* and *Panicum aciculare* can also dominate lower areas in backdunes.

Of greater interest to this study were species that were correlated with changes in elevation, as this might suggest species that either influence or respond to sand movement and may be more important for climate change responses. Despite the significant effects of change in elevation on ordination scores, relatively few species showed strong positive or negative correlations with change in elevation over 2011–13. *Uniola paniculata* did show an increase in abundance as dunes lost elevation on foredunes. This was somewhat surprising, as *U. paniculata* has been implicated as a dune builder. However, on St George Island, *Uniola* is also dominant on older foredunes that are starting to erode as they become blocked by newer dunes forming towards the beach, which may cause a net negative association with elevation. *Polypremum procumbens* was significantly associated with increases in elevation in interdunes, *Polygonum punctatum*, a wetland specialist in dunes, was associated with decreases. These species are good indicators of low and high dune areas respectively, but both are relatively infrequent and unlikely to have a large role in reciprocal interactions between vegetation and dune morphology.

This study demonstrates that changes in elevation in coastal areas are correlated with changes in the associated plant community, which is consistent with previous studies such as Moreno-Casasola (1986) and Wolner *et al.* (2013). Further, it demonstrates that these relationships change with dune habitat, which predicts how effects of climate change will vary spatially. Foredune vegetation is very much affected by the more variable and disturbed nature of the dynamic sand movement near the shore. Plants that help build or sustain dunes will be particularly important. Interdunes are low, wet and less variable in elevation and there are suggestions that plants may play a significant role in actually preventing dunes from building (Ehrenfeld 1990; Stallins 2005; Wolner *et al.* 2013). Backdunes retain dune and trough associated variation in elevation, but are much more stable than other areas. The influence of long-lived species, including woody species, suggests that these areas may also be the result of longer-term successional processes. Overall, the interactions between dune geomorphology and their associated communities are complex and vary at different spatial scales.

Just as relationships between dune geomorphology and vegetation vary with dune habitat, they are also likely to change across different spans of years. The years used in this study were particularly quiescent; the closest storm, Tropical storm Debby, passed more than 100 miles south of St George Island in 2012. It will be important to follow these same patterns during extremes of storms and droughts that are known to have major effects on dune geomorphology and vegetation (see Hesse and Simpson 2006; Miller *et al.* 2010).

## Conclusions

Previous studies have documented correlations between environmental traits and vegetation, primarily in foredunes. However, few studies have correlated disturbance in dunes with plant community composition and no prior studies have investigated how the effect of disturbance varies at different spatial scales. This study used a unique long-term data base from a barrier island in the northern Gulf of Mexico to correlate the vegetation patterns with dune habitat, elevation and the change in elevation from 2011 to 2013. Generalized linear models suggest that both elevation and change in elevation (disturbance) do affect the vegetation, but that these effects differ among foredunes, interdunes and backdunes. A particularly notable result is that, while interdunes are subject to only minor disturbances, these can have significant effects on the plant community, perhaps because of changes in the hydrology of this lower-lying habitat. Overall, this study provides a better view of the links between dynamic dune geomorphology and the plant community, which may be important for predicting future effects of climate change on coastal ecosystems.

## Sources of Funding

This work was funded in part by an award from the National Science Foundation (DEB 1050469) to T.E.M. and by funds from Florida State University.

## Contributions by the Authors

T.E.M. is responsible for all the organization of the fieldwork, data management, analyses and writing for this study.

## Conflicts of Interest Statement

None declared.
